# Technical note: Practical application of post-mortem mechanical stimulation of skeletal muscle, a field study

**DOI:** 10.1007/s00414-022-02873-3

**Published:** 2022-08-13

**Authors:** H. Stigter, T. Krap, T. Gelderman, L. Dijkhuizen, WLJM Duijst

**Affiliations:** 1 Faculty of Law and Criminology, Maastricht University, Minderbroedersberg 4-6, 6211 LK Maastricht, The Netherlands; 2 Ars Cogniscendi Foundation for Legal and Forensic Medicine, Wezep, The Netherlands

**Keywords:** Post-mortem interval, Mechanical stimulation, Supravital, Excitability, Muscle contraction, Learning period, Musculus biceps brachii, Musculus brachioradialis

## Abstract

**Background:**

Estimation of the post-mortem interval (PMI) is a crucial aspect in crime scene investigation. PMI is defined as the time between the moment of death and the moment of finding the dead body. A combination of methods for estimating the PMI in forensic casework is internationally mostly used. Supravital muscle reaction (SMR) is one of those methods. SMR is an idiomuscular contraction and can be provoked by mechanical stimulation.

**Objectives:**

A field study was carried out with the aim to investigate whether a reflex hammer can be used as tool for triggering an idiomuscular contraction and, furthermore, to determine if a learning period has to be taken into account by a forensic physician for appropriate application of a reflex hammer to trigger SMR.

**Methods:**

From January 2017 to January 2022, four forensic physicians used this SMR by mechanically stimulating the musculus brachioradialis and musculus biceps brachii. In total, 332 cases were included with a PMI less than 24 h. The cases were divided in chronological clusters of 20 cases. The ratio of the number of positive SMR versus the total number stimulations per forensic physician was used as a measure of accuracy of a reflex hammer for triggering SMR. The distribution of the data was analyzed by comparing the clusters in chronological order to assess whether a learning curve applies.

**Results:**

In 55.7%, a muscle reaction could be provoked by mechanical stimulation. Comparable outcome of SMR between the participating physicians was observed after 40 stimulations.

**Conclusion:**

A reflex hammer is usable for provoking SMR. A learning period has to be taken in to account during the first forty cases per forensic physician.

## Introduction

As a part of the postmortem investigation the forensic physician in the Netherlands often has to estimate the post-mortem interval (PMI) [1]. The PMI represents the time between the moment of death and the moment of finding the dead body. Henssge and Madea recommend a compound method for estimating the PMI in forensic casework [2–4]. Supravital muscle reaction (SMR) is one of the suggested methods as part of the compound method. SMR is an idiomuscular contraction and can be provoked by mechanical stimulation. The reaction is dependent on the post-mortem storage of glycogen and adenosine triphosphate (ATP) and disappears under the influence of irreversible changes in the internal environment due to anaerobic glycolysis and decomposition [5]. SMR can be triggered by a single forceful vertical hit on the muscle’s belly [2–8]. Warther et al. used a steel chisel to mechanically stimulate the belly of the musculus biceps brachii and the musculus quadriceps femoris bilaterally. The study population used by Warther et al. consisted of subjects that had died in hospital or arrived dead in hospital after out-of-hospital witnessed sudden death. The forensic physician in the Netherlands investigates deceased both in- and outside health care institutions. Although the study population used by Warther et al. consisted of in and out-of-hospital deaths, the latter group was immediately transferred to a forensic institute for examination. Therefore, the population used by Warther et al. does not represent the broader population of deceased being examined by the forensic physician in daily work.

A reflex hammer is part of the standard diagnostic tools of the forensic physician. It is mainly used for basic health care of detainees. A reflex hammer could be used to trigger SMR. However, when changing the method, in accordance with the Daubert criteria, it must first be demonstrated that these changes are valid. But, to determine the validity of the newly introduced method it is, also in accordance with the Daubert criteria, crucial to know whether the operator is able of applying the method. The aim of this study was, therefore, to investigate whether a post-mortem contraction of skeletal muscle can be observed visually or manually, after hitting the muscle with a reflex hammer in case of in-hospital deaths, out-of-hospital deaths that are transferred to hospital for further investigation and out-of-hospital deaths that are examined at the place where the body was found. In addition, it was investigated whether a learning period applies when a forensic physician applies this method in practice.

## Methods

**Fig. 1:**
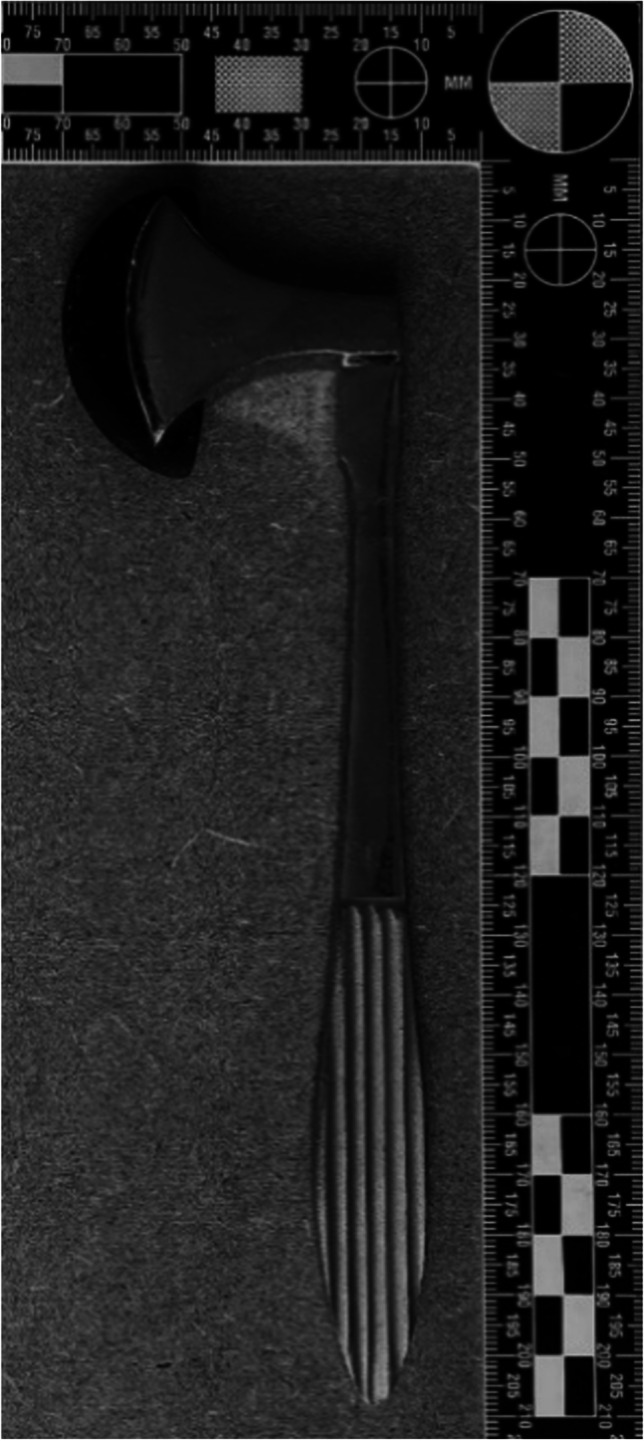
Reflex hammer; model Berliner, 132 g

**Fig. 2:**
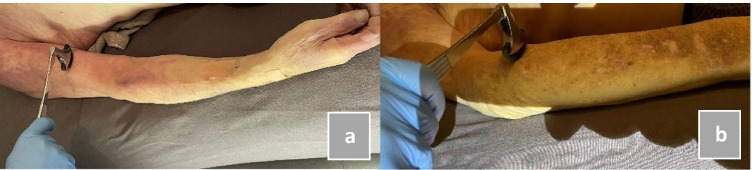
Location of hit with reflex hammer: **a** belly musculus biceps brachii, **b** proximal part musculus brachioradialis

In the period of January 2017 to January 2022, four forensic physicians from two different regions in the Netherlands used a reflex hammer to trigger SMR. In this study, a standard reflex hammer was used for triggering idiomuscular contraction (see Fig. 1). Since skeletal muscles of the upper extremities are generally more easily accessible than muscle groups of the lower extremities, the musculus biceps brachii and the musculus brachioradialis were stimulated. Mechanical stimulation of the belly of the musculus biceps brachii and the proximal part of the musculus brachioradialis was achieved by a single vertical hit (see Fig. 2).

The population consisted out of 332 deceased, of which 186 were male and 146 female, with a mean age of 69.6 ± 41.4 years, concerning deaths both in- and outside health care institutions (see Table 1). Inclusion criteria were adult with a PMI of less than 24 h based on information derived from a medical file or police investigation. Exclusion criteria were age below 18 years, severe injury, and bodies with signs of decomposition with a total decomposition score ≥ 7 on the Gelderman scale [9]. In 26 out of 332 cases, the place of examination of the deceased could not be traced due to change of registration software. In 122 out of 306 cases (39.9%), the post-mortem investigation was performed outside a health care institution at the place where the body was found.

**Table 1:** Population characteristics per forensic physician

Forensic physician	Mean age (years)	Male	Female	Total bodies
1	70.5 ± 39.8	49 (49.0%)	51 (51.0%)	100
2	69.3 ± 40.2	61 (61.0%)	39 (39.0%)	100
3	70.9 ± 44.6	31 (58.5%)	22 (41.5%)	53
4	68.2 ± 43.0	45 (57.0%)	34 (43.0%)	79
Total	69.6 ± 41.4	186 (56.0%)	146 (44.0%)	332

**Fig. 3:**
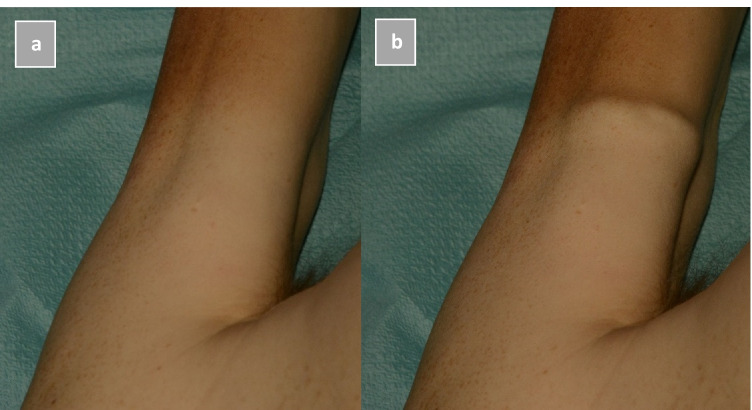
SMR of musculus biceps brachii; **a** before stimulation, **b** after stimulation

In an attempt to prevent a possible false positive interpretation, due to an initially bumpy surface, the surface of the musculus biceps brachii and/or musculus brachioradialis was manually examined before mechanical stimulation. In addition, to avoid false interpretation of outcome due to preceding provocation, stimulation was not repeated. The outcome of SMR was considered positive (present) if a muscle contraction was detectable in at least one of the two stimulated muscles, either visibly or manually through palpation immediately after mechanical stimulation (see Fig. 3). If neither a visible nor palpable reaction was detected, the result was registered as negative (absent). All results were registered in the Dutch national register of forensic medicine (Formatus), which was used as a data resource.

The ratio of the number of bodies with a positive SMR versus the total number of bodies stimulated per forensic physician was used as a measure of accuracy of the reflex hammer for triggering SMR.

The cases per forensic physician were categorized in chronological order in clusters of a maximum of 80 bodies per cluster. Cluster 1 (cases 1–80) being the cases first performed followed by cluster 2 (cases 81–160), and so on. The percentage present and absent outcome were calculated per physician for each cluster and plotted in a boxplot. To determine if the participating forensic physicians had to conduct a learning for appropriate application of SMR by using a reflex hammer, the distribution of the data was studied by comparing the clusters in chronological order, taking in consideration the expected percentage of positive SMR (16.7%) based on the data provided by Warther et al. [5].

## Results

Of all the 332 included cases, the actual PMI was less than 8 h and therefore fell within the 24-h inclusion criterium. In 185 of the 332 included cases (55.7%), SMR could be provoked following mechanical stimulation with a reflex hammer (Table 2). Different outcomes of SMR after mechanical stimulation were noticed. In some cases, not every mechanically stimulated muscle from the same body showed a contraction. Assessment of SMR of the musculus biceps brachii after mechanical stimulation was difficult for especially elderly people.

**Table 2:** Outcome of SMR per tested body per forensic physician

Forensic physician	Muscle contraction present	Muscle contraction absent	Total
1	67 (67.0%)	33 (33.0%)	100
2	48 (48.0%)	52 (52.0%)	100
3	24 (45.3%)	29 (54.7%)	53
4	42 (53.2%)	37 (46.8%)	79
Total	185 (55.7%)	147 (44.3%)	332

**Fig. 4:**
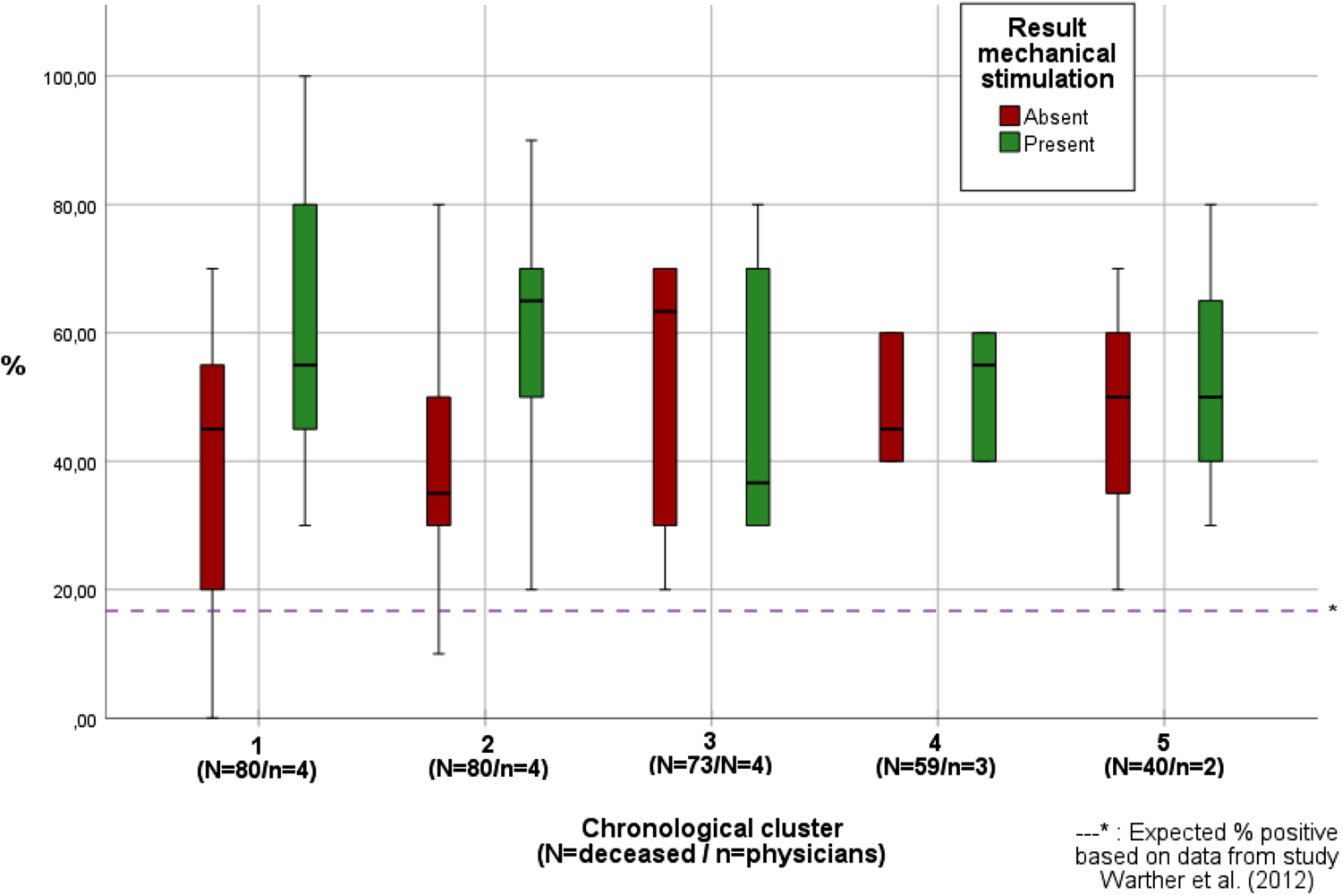
Outcome of SMR per cluster of bodies (% absent versus % present)

The variation in outcome of SMR in the first 2 clusters was larger when compared to clusters 3, 4, and 5 (see Fig. 4).

## Discussion

In the current study, relatively more bodies (55.7% of 332 cases) showed SMR following mechanical stimulation by using a reflex hammer compared to the results of the study by Warther et al., where SMR was positive in 16.7% of 270 cases by using a steel chisel [5]. This difference in results could be explained by a difference in PMI. In the current study, in all the included cases, the PMI was less than 8 h while in the study by Warther et al. in 240 out of 270 cases, the PMI was at least 8 h, and in 60 of those 240 cases, the PMI was at least 14 h [5]. Therefore, based on the PMI, a higher number of positive SMR after mechanical stimulation could be expected in the current study. In order to validate the use of a reflex hammer for provoking SMR for estimating the PMI, it is necessary to have a study population with a range of PMI comparable to the population used by Warther et al.

Another difference between this study and the study by Warther et al. that has to be considered deals with the place of examination. In the study by Warther et al., all bodies were examined in hospital, meaning that persons who passed away after out-of-hospital sudden death were transferred to hospital. To transfer a body, it is necessary to manipulate the body. Manipulation of a body, for instance by pulling an arm, implicates movement of skeletal muscle and accordingly use of energy by these muscle cells. Loss of energy has a negative effect on SMR. In the current study, the place of death was registered in 306 cases. In 39.9% of these 306 cases, the bodies were examined at the place of death. So, these bodies were not manipulated because transfer to hospital or a forensic institute was not the case. Hence, loss of energy in skeletal muscle should have been less, and accordingly, more bodies showing positive SMR could be expected.

Other influencing factors that can alter the outcome of provoking SMR have to be taken into account. Warther et al. mentioned that a possible limitation of the method was that due to body-related factors as obesity, infectious diseases, and fluid overload on an intensive care unit, SMR was hardly observed [5]. These possible influencing factors are likely different for the two studied populations, contributing to the observed difference in positive outcome.

As could be expected from the average PMI in this study, a higher number of bodies with SMR were observed for all four participating forensic physicians. In this regard, it should be taken into account that, at the start of this study, the level of experience in handling a reflex hammer for mechanical stimulation of skeletal muscle might have differed between the four forensic physicians. However, no specific training on using a reflex hammer for this purpose seems to be necessary since all forensic physicians obtained positive outcomes during the first 20 investigations.

The variation in outcome of SMR decreased during the period of investigation, resulting in a more comparable outcome of SMR among observers. Although variation can be expected among deceased within the studied population, it is not expected that the variation for 40 to 80 deceased per cluster, and the decrease in difference in variation from cluster 2 to 3, can be solely explained by chance, especially not when taking in to account the low percentage of expected positive outcomes based on the data of Warther et al. [5]. Therefore, this finding substantiates the hypothesis that the forensic physician learns to better perform the test and interpret the outcome.

Warther et al. confirmed the upper limit of 13 h post-mortem based on mechanical stimulation of the musculus biceps brachii and the musculus quadriceps femoris, muscles that are relatively near the central part of the body [5]. Relaxation of muscles after contraction is a process based on the conversion of ATP present in muscle cells in adenosine diphosphate (ADP). ATP itself is reproduced by conversion of glycogene into lactate. In case of death, the level of energy in cells will drop and accordingly the level of glycogene and ATP. Due to ATP deficiency, muscle cells cannot relax, causing the muscle to stiffen, rigor mortis [10]. Thus, like SMR, rigor mortis is also based on the storage of glycogen and ATP in muscle cells after death. A low body temperature slows down cell metabolism, leading to a decrease in energy consumption and results in ATP being longer available. Kobayashi et al. showed a low rate of progress of rigor mortis with lower ambient temperatures. The rate of body cooling is higher in more peripheral located muscles than central, more proximal located muscles [11]. Compared to the musculus biceps brachii and the musculus quadriceps femoris, the musculus brachioradialis is located farther away from the trunk of the body, leading to the hypothesis that the upper limit of PMI could be higher after stimulating skeletal muscle located more peripheral from the trunk due to prolonged availability of ATP. Therefore, besides more research on different populations, it is also advised to study the SMR of more anatomical regions.

## Conclusion

The results show that a reflex hammer is usable to trigger SMR by mechanical stimulation of skeletal muscle of the upper extremities. However, with regard to the associated PMI, more research has to be conducted for validation of the upper limit when using a reflex hammer. Comparable outcome of SMR between the participating physicians was observed after 40 stimulations, leading to the conclusion that a learning period has to be taken in to account during the first forty files per forensic physician.
